# A structural discovery journey of streptococcal phages adhesion devices by AlphaFold2

**DOI:** 10.3389/fmolb.2022.960325

**Published:** 2022-08-19

**Authors:** Adeline Goulet, Raphaela Joos, Katherine Lavelle, Douwe Van Sinderen, Jennifer Mahony, Christian Cambillau

**Affiliations:** ^1^ Laboratoire d’Ingénierie des Systèmes Macromoléculaires (LISM), Institut de Microbiologie, Bioénergies et Biotechnologie, CNRS, Aix-Marseille Université, UMR7255, Marseille, France; ^2^ School of Microbiology, University College Cork, Cork, Ireland; ^3^ APC Microbiome Ireland, University College Cork, Cork, Ireland; ^4^ AlphaGraphix, Formiguères, France

**Keywords:** bacteriophage, Streptococcus, AlphaFold2, phage-host interactions, carbohydrate-binding module, receptor-binding protein

## Abstract

Successful bacteriophage infection starts with specific recognition and adhesion to the host cell surface. Adhesion devices of siphophages infecting Gram-positive bacteria are very diverse and remain, for the majority, poorly understood. These assemblies often comprise long, flexible, and multi-domain proteins, which limits their structural analyses by experimental approaches such as X-ray crystallography and electron microscopy. However, the protein structure prediction program AlphaFold2 is exquisitely adapted to unveil structural and functional details of such molecular machineries. Here, we present structure predictions of whole adhesion devices of five representative siphophages infecting *Streptococcus thermophilus*, one of the main lactic acid bacteria used in dairy fermentations. The predictions highlight the mosaic nature of these devices that share functional domains for which active sites and residues could be unambiguously identified. Such AlphaFold2 analyses of phage-encoded host adhesion devices should become a standard method to characterize phage-host interaction machineries and to reliably annotate phage genomes.

## 1 Introduction

The release of the powerful AlphaFold2 (AF2) software in mid-2021 revolutionised structural biology (Jumper et al., 2021a; Jumper et al., 2021b; Tunyasuvunakool et al., 2021). AF2 makes it possible to accurately predict the structures of proteins and their complexes (Evans et al., 2021). Furthermore, an index called predicted local distance difference test (pLDDT) provides an estimate of the prediction accuracy along the protein chain, from 0 to 100 (best). In practice, pLDDT values over 80–90 compare to average resolution X-ray structures. We reason that AF2 may be an appropriate method to study host adhesion devices of bacteriophages (phages) that are often assembled from long, flexible, and multi-domain proteins, which limits their structural analyses by experimental approaches such as X-ray crystallography and electron microscopy. Recently, we applied this to the study of different adhesion devices of the *Oenococcus oeni* phages OE33PA and Vinitor 162, using a preliminary version of AF2 that did not include the multimer option (Goulet and Cambillau, 2021).

Phages infecting the dairy bacterium *Streptococcus thermophilus* have been the focus of significant research attention in recent years due to the threat they pose to global industrial dairy fermentations (Mahony et al., 2012; McDonnell et al., 2016; McDonnell et al., 2017; Lavelle et al., 2018b). Recently, we analysed the adhesion device of *S. thermophilus Moineauvirus* and *Brussowvirus* siphophages (formerly termed the *cos* and *pac* phages, respectively) using HHpred and identified several carbohydrate-binding modules (CBMs) in two conserved siphophage tail components termed the distal tail protein (Dit) and the tail associated lysin (Tal) (Lavelle et al., 2020). Host binding studies using these CBMs confirmed their functionality as well as their specificity for each phage’s host (Lavelle et al., 2020). Unexpectedly, we also discovered a third ORF, located downstream of the Tal-encoding gene, encoding a previously unidentified receptor-binding protein (RBP).

While the structure of recombinantly expressed CBMs, RBPs or some complete adhesion devices may be determined by X-ray crystallography (Sciara et al., 2010; Veesler et al., 2012; Dieterle et al., 2017), the adhesion device of *Moineauvirus* and *Brussowvirus* as a whole is beyond the possibilities of these techniques. Indeed, many of these phages possess large Tal proteins (∼800–2,500 amino acids) as well as long and flexible extensions, which have been observed by negative staining electron microscopy (nsEM) (Szymczak et al., 2017; Lavelle et al., 2018a; Hanemaaijer et al., 2021). Furthermore, Dit proteins of several siphophages have been reported to harbour CBMs (in which case they are termed evolved Dits), and the presence of such CBMs at the extremity of long and flexible linkers prevent them from being analysed *in phago* (Dieterle et al., 2017). Therefore, our nsEM 3D reconstruction of the *Moineauvirus* phage STP1 adhesion device showed well-resolved density only for the Dit central hexameric ring and the Tal trimeric N-terminal domain (Kanamaru et al.,2002) with partly-defined densities at its periphery accounting for six trimeric RBPs (Lavelle et al., 2020).

Here, we applied a structure prediction approach to a carefully chosen set of five phages belonging to the *Moineauvirus* and *Brussowvirus* genera with the latest version of AlphaFold2 multimer (as of January 2022) (Evans et al., 2021). In this manner, we were able to assemble complete structural models of the Dit-Tal assembly as well as a model of the RBPs. These models reveal that *S. thermophilus* phages use multiple CBMs, which act together with the *bona fide* RBPs, to bind to their host-specific cell wall polysaccharide (CWPS) (Mahony et al., 2020). Noteworthy, these CBMs are LEGO-like assembled giving rise to a structural, and likely functional, variety of *S. thermophilus* phages’ adhesion devices. In particular, the different CBM combinations identified in the Tals are built from a variable number of similar modules. Finally, the method described here makes it possible to perform a precise annotation of phage adhesion devices, far beyond the reach of other methods such as HHpred (Zimmermann et al., 2018).

## 2 Materials and methods

### 2.1 Phage selection

Five *S. thermophilus* phages were selected for analysis in this study that represent both the *Moineauvirus* and *Brussowvirus* genera. Moineauviruses DT1 (Lamothe et al., 2005), STP1 (Lavelle et al., 2018b) and *Brussowviruses* 9851 (McDonnell et al., 2017), TP-778L (Ali et al., 2014) and SW13 (Lavelle et al., 2018a) were analysed in this study. The Genbank accession numbers for the phages are as follows: DT1 (NC_002072.2), STP1 (MF580773.1), 9851 (KY705284.1), TP-778L (NC_022776.1) and SW13 (MH892362.1).

Phage 9851, isolated from a dairy fermentation in France, infects *S. thermophilus* strain ST64985 (McDonnell et al., 2017). Phage TP-778L is an induced (pro)phage of strain SK778, and it can be propagated on host strain B106 (Ali et al., 2014). Phage DT1, isolated from a Mozzarella whey in Canada, infects *S. thermophilus* SMQ-301 (Tremblay and Moineau, 1999). Phage STP1, isolated from an Irish cheese whey, infects *S. thermophilus* UCCSt102 (Lavelle et al., 2018b). Phage SW13, isolated from a Turkish dairy facility, infects *S. thermophilus* UCCSt50 (Lavelle et al., 2018a).

### 2.2 Protein structure predictions and topological model assembly

Although HHpred predictions were reported in a previous study, we performed HHPred analyses on the Tals to obtain up-to-date information of their domain composition (Zimmermann et al., 2018). We used a Colab’s notebook (https://colab.research.google.com/github/deepmind/alphafold/blob/main/notebooks/AlphaFold.ipynb#scrollTo=XUo6foMQxwS2) to perform the predictions. To note, this notebook does not use PDB templates (as do “true” AlphaFold2 servers), thereby providing a totally naive structure prediction. Furthermore, this ColabFold allows the modeling of homo-multimers. Due to memory limitations, long sequences had to be split in sequence stretches with considerable overlap for later assembly. In a first pass, we ran structure predictions for monomers in order to determine sensible stretch boundaries to be assembled in trimers (Tal, RBP) or hexamers (Dit). The number of residues in the multimeric stretch predictions had to be less than 1,400 residues. Moreover, we predicted structures of stretches with overlapping segments to allow full-length assembly of the full-length multimers using *Coot* (Emsley et al., 2010). The pLDDT values that are stored in the pdb file as B-factors, were plotted using Excel (Supplementary Figures S4A,B). The final predicted domain structures were submitted to the Dali server (Holm, 2020) to identify the closest structural homologs in the PDB. In order to assemble topological models of Dit-Tal assemblies, we used the *Coot* option “SSM Superpose” to superimpose individual domains onto the corresponding ones of the lactococcal phage p2 adhesion device (Sciara et al., 2010). Sequence alignments were performed with Multalin (Corpet, 1988) and ESPript (Gouet et al., 2003). Visual representations of the structures were prepared with ChimeraX (Pettersen et al., 2021).

## 3 Results

We selected five representative *S. thermophilus* phages for detailed structural analysis based on three criteria: 1) they have been isolated in geographically distinct locations, 2) they possess unique host ranges, and 3) they exhibit different adhesion devices according to their sequences. Furthermore, based on a recent phylogenetic analysis of dairy streptococcal phages (Hanemaaijer et al., 2021), the genomes of the selected phages are overall distinct. Among these, three are members of the *Moineauvirus* genus (DT1, STP1, 9851), and two are members of the *Brussowvirus* genus (SW13, TP-778L). Phage 9851 was isolated from a dairy fermentation in France, and it infects *S. thermophilus* strain ST64985 (McDonnell et al., 2017). Phage TP-778L is an induced (pro)phage of strain SK778, and it can be propagated on host strain B106 (Ali et al., 2014). Phage DT1 was isolated from a Mozzarella whey in Canada, and it infects *S. thermophilus* SMQ-301 (Tremblay and Moineau, 1999). Phage STP1 was isolated from an Irish cheese whey, and it infects *S. thermophilus* UCCSt102 (Lavelle et al., 2018b). Phage SW13 was isolated from a Turkish dairy facility, and it infects *S. thermophilus* UCCSt50 (Lavelle et al., 2018a).

### 3.1 Predicted structures of Dits

**FIGURE 1:**
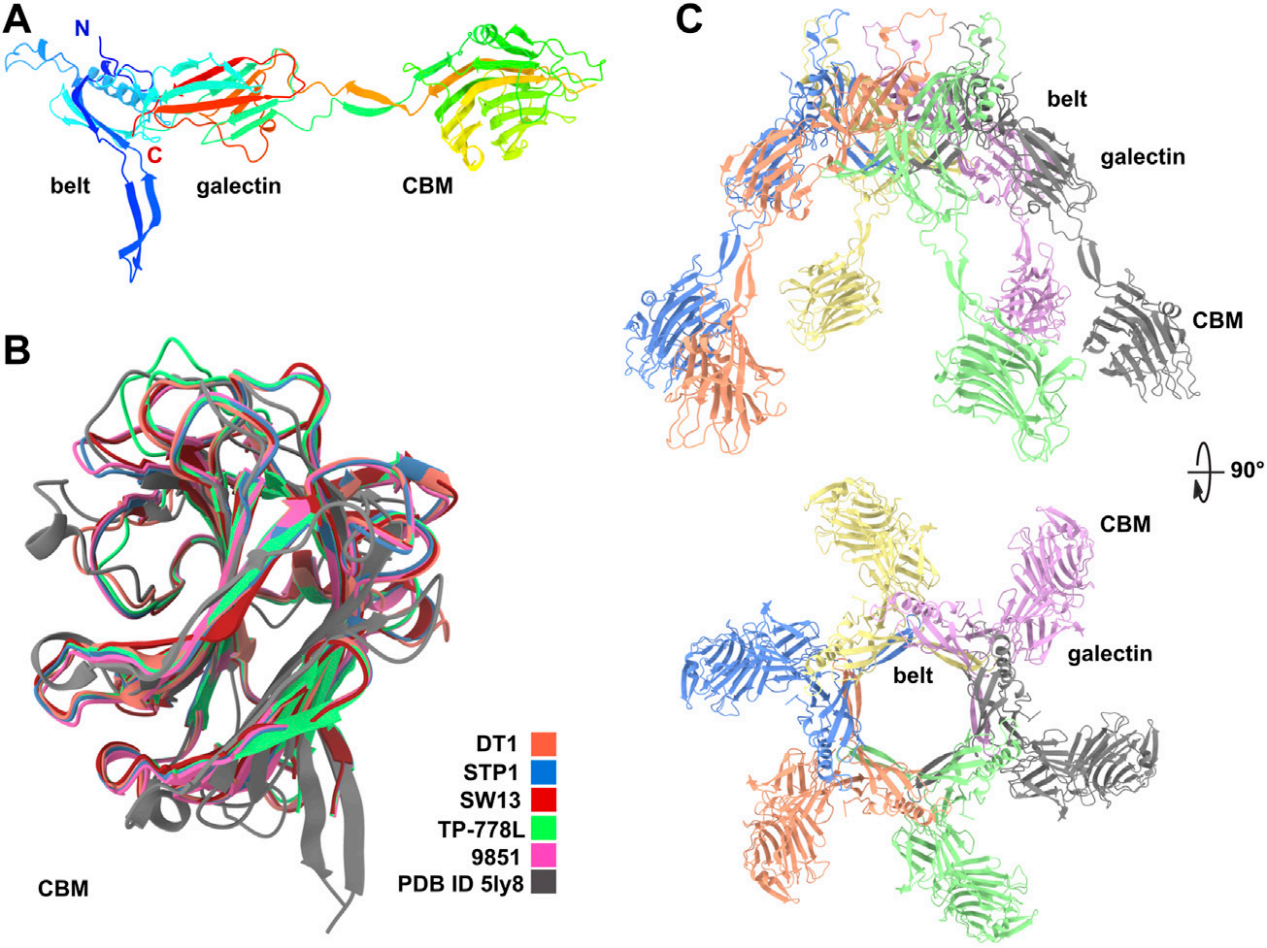
Predicted structure of Dits. **(A)** Ribbon representation of TP-778L Dit monomer with its three domains, belt, galectin and CBM (rainbow colored). **(B)** Ribbon representation of the superimposition of the five Dit CBMs together with the Dali’s best hit [PDB ID 5ly8; *Lactobacillus* phage J-1 Dit CBM (Dieterle et al., 2017)]. **(C)** Ribbon representation of the TP-778L Dit hexameric assembly (orthogonal views). The top loops are poorly predicted and are suspected to interact with the most distal major tail protein (MTP) ring (colored by chain).

Dit proteins can be divided into two domains corresponding to the N- and C-terminal parts of the polypeptide chain. The N-terminal domain, called the belt, is composed of two β−sheets, a β−hairpin, and an α−helix. The C-terminal domain, called the galectin, is a two β−sheet structure, similar to a galectin domain (Veesler et al., 2010). Of note, this galectin domain can be absent in some Dits, such as in phage Lambda, or can be replaced by an OB-fold domain, such as in phage T5 (Flayhan et al., 2014). As mentioned above, Dits possessing CBM insertions in the galectin domain are called evolved Dits (Dieterle et al., 2017). In phage tails, six Dit monomers assemble as a ring allowing DNA passage. The ∼500 amino acid long Dit proteins of *S. thermophilus* phages are classified as evolved since they contain a CBM, inserted within the galectin domain, clearly identified by HHpred (Lavelle et al., 2020). AF2 predictions of the Dit from the five *S. thermophilus* phages yielded very similar topologies. The predicted belt and galectin domains are structurally close to those whose structures have previously been determined experimentally, as exemplified by the finding that the *Bacillus subtilis* phage SPP1 Dit was returned as a hit by the Dali server (Holm, 2020) (Figure 1A; Supplementary Table S1). The ∼250 amino acid long CBMs are inserted in the galectin domain in between two long linkers, and share similar folds (Figure 1B). The Dali server returned the same hit for each of these CBMs, corresponding to the CBM of the evolved Dit from *Lactobacillus casei* BL23 phage J-1 (Dieterle et al., 2017) (Supplementary Table S1). In the predicted hexameric assemblies of these evolved Dits, the six CBMs project quite prominently (∼60 Å) from the belt ring (Figure 1C), which would enable them to interact with their glycan receptor located on the host cell surface. Interestingly, although these CBMs share a common fold, they can be classified in two groups based on sequence differences: the first group comprises the Dit of DT1, STP1, and 9851, and the second group comprises the Dit of SW13 and TP-778L (Supplementary Figures S1A,B). These sequence variations within a common fold suggest that each CBM may be adapted to recognize host-specific CWPS receptors.

### 3.2 Predicted structures of Tals

**FIGURE 2:**
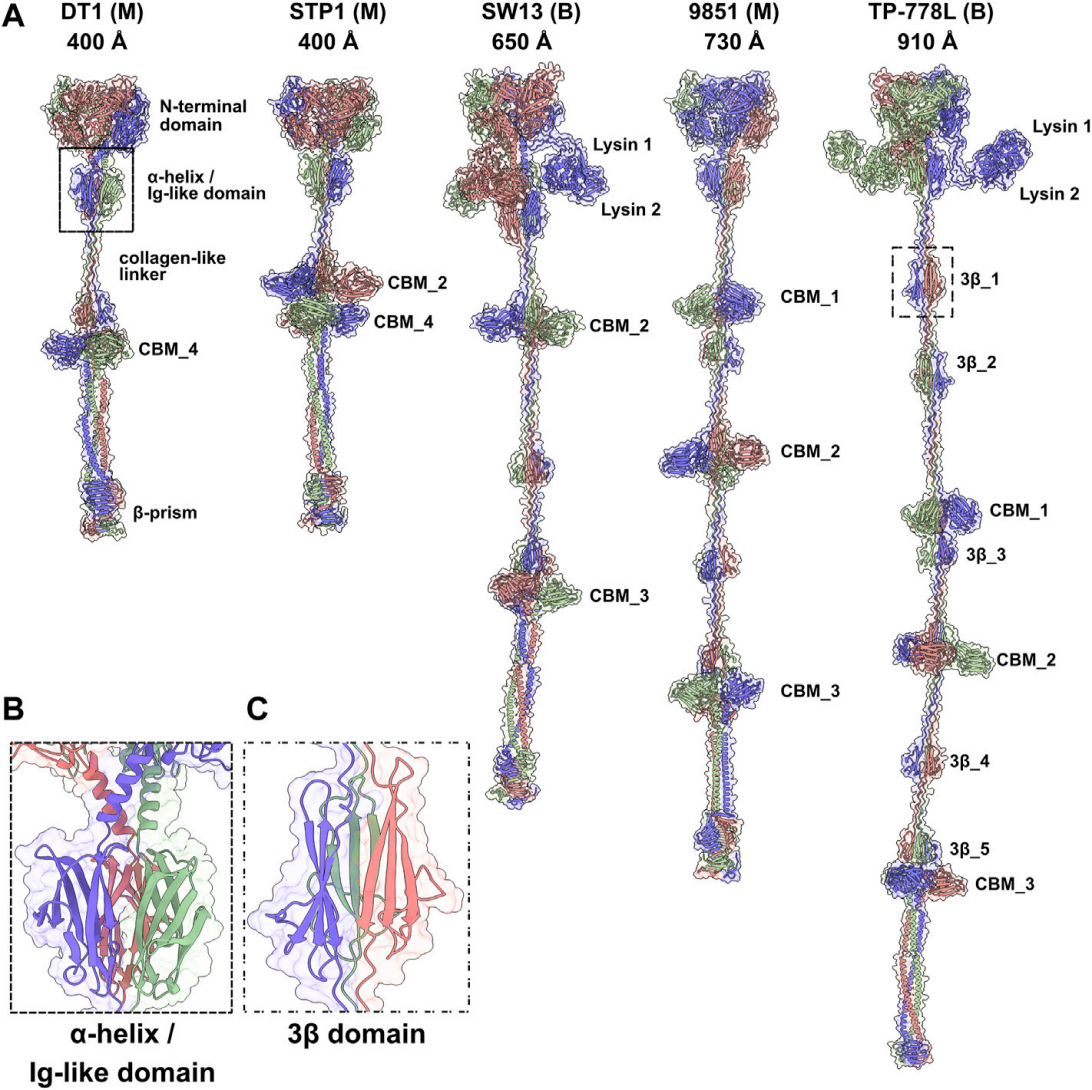
Predicted structures of Tals. **(A)** Ribbon and transparent surface representations of trimeric Tals (colored by chain) from the five *S. thermophilus* phages. The various domains are indicated as listed in Table 1. Junctions between N-terminal structural domains and C-terminal extensions were not reliably predicted, therefore we did not join them in our structural models. **(B)** Close-up view on the α-helix and Ig-like domain that follow the Tal N-terminal structural domain. **(C)** Close-up view on a 3β domain.

Tals of siphophages are composed of an N-terminal structural domain of ∼350–400 amino acids (Sciara et al., 2010). In many phages, this domain is followed by an extension that is believed to play a role in CWPS/peptidoglycan degradation, e.g., the *Lactococcus lactis* P335 phage TP901-1 (Stockdale et al., 2013), or host binding, e.g., the *B. subtilis* and *Escherichia coli* phages SPP1 and T5 (Sao-Jose et al., 2006; Linares et al., 2020). Tal extensions of the five phages analysed in this study are of variable lengths, ranging from 914 amino acids in DT1 to 1,981 amino acids in TP-778L (Table 1). Consistent with their number of amino acids, the length of Tal predicted structures varies from 400 Å for the shortest (DT1, STP1) to 910 Å for the longest (Figure 2A).

**TABLE 1:** Tai domain boundaries in predicted structures (M: *Moineauvirus*; B: *Brussowvirus*; ^§^: PDB ID; Z score).

	DTl (M)	Dali hit^§^	STPl (M)	Dali hit^§^	SW13 (B)	Dali hit^§^	9851 (M)	Dali hit^§^	TP-77SL(B)	Dali hit^§^
N-termin.al structural domain										
Sub-domains 1-3					1-239				1-237	
Lysin 1					273-450	3fi7;14 .8			273-445	3fi7;15.0
linker					451-462				446-461	
Lysin 2					463-619	5d74;21.0			462-622	5d74;21.3
Sub-domains 1-4 Sub-domain 4	1-384 -	3cdd;l6.8	1-384 -	3cdd;l6.3	- 659-789	2x8k;20.2	1-379 -	3cdd;l6,5	- 656-790	3gs9;17.5
gap										
C-termin.al extension.										
α-helix	385-401		385-401		790-806		388-400		792-809	
Ig-like domain	402-490	6grs;9.8	402-490	6grs;9.5	807-893	6grt;9.l	402-490	6grs;9.6	810-897	6grs;9.8
linker-β	491-512		491-504		894-911		491-515		898-917	
Module 3β _1									918-977	2rbl;4.8
linker-β									978-992	
module 3β_ 2									993-1051	2rbl;4.2
linker-β									1052-1084	
CBM_1							516-675	5ggf;l6.9	1085-1252	5ggf;l6.l
linker-β							676-688		1253-1257	
module 3β_ 3							688-740	5e7t;8.9	1258-1306	5e7t;8.9
linker-β							741-742		1307-1325	
CBM_2			505-736	5e7t;28.6	912-1105	5e7t;13.3	743-961	5e7t;l4.8	1326-1530	5e7t;l5.2
linker-β			737-741		1106-1147		962-989		1531-1559	
module 3β _4					1148-1193	5e7t;7.7	990-1040	5e7t;8.2	1560-1606	5e7t;7.0
linker-β					1194-1214		1041-1055		1607-1625	
module 3β_ 5	513-561	5e7t;9.0			1215-1262	5e7t;8.6	1055-1109	5e7t;9.4	1626-1675	5e7t;8.5
linker-β	562-566				1263-1284		1110-1119		1677-1682	
CBM_3	-	-	-	-	1285-1423	3pb6;10.8	1120-1267	3pb6;12.l	1683-1844	3p6b;l2.6
CBM_4	567-739	5w6h;l5.0	742-916	2zey;l4.8	-	-	-	-	-	-
α-helix	740-817		917-1005		1424-1519		1276-1355		1845-1940	
β-prism/β-helix	818-914	3pqh;7.0	1006-1092	4bxq;6.2	1520-1609	7chu;6.0	1358-1452	6u9g;6.4	1941-1981	4bxr;7.2

Tals from several *S. thermophilus* phages have previously been examined by HHpred (Lavelle et al., 2020). These analyses revealed the presence of CBMs within Tal extensions. In the current study, an updated HHpred analysis of Tal from each of the five selected phages identified catalytic domains in the N-terminal structural domain as well as CBMs in the Tal extension (Supplementary Figures S2A,B).

#### 3.2.1 The Tal N-terminal domain can be functionalized

Tals are trimeric proteins that stack against the Dit hexameric ring. The Tal N-terminal domain resembles the N-terminal domain of gp27, the puncturing device of the myophage T4 (Kanamaru et al., 2002), and the N-terminal domain of the T6SS VgrG protein (Veesler and Cambillau, 2011). This domain assembles four sub-domains, numbered 1–4, along the sequence. Sub-domain 1 is formed by two β-sheets, sub-domains 2 and 3 are constituted by one or two helices stacked against a β-sheet, and sub-domain 4 is formed by two β-sheets. Sub-domains 1 and 4 exhibit structural similarities and form a pseudo-hexameric ring in the Tal trimer, thereby allowing an efficient packing against the Dit hexamer. Some Tals comprise only this gp27-like structural domain, as in *Skunavirus* (Sciara et al., 2010), while the majority contain an extension of varying lengths after sub-domain 4 (Hanemaaijer et al., 2021).

**FIGURE 3:**
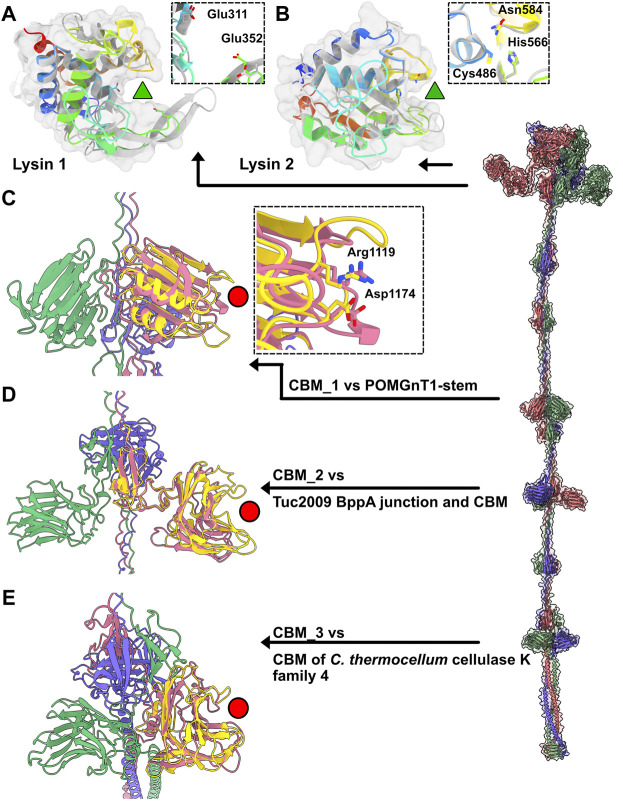
Catalytic and binding domains of phage TP-778L Tal. **(A)** Ribbon and transparent surface representation of TP-778L lysin_1 superimposed to the catalytic domain of *L. monocytogenes* autolysin Auto (Lmo1076) (PDB ID 3fi7; Table 1), a N-acetyl glycosaminidase (Bublitz et al., 2009). The catalytic residues of Auto occupied the same position as Glu311 and Glu352 of lysin_1 (residues shown as sticks). The active site cavity is indicated by a green triangle. Lysin_1 ribbon is rainbow colored, that of Auto is grey). **(B)** Ribbon and transparent surface representation of TP-778L lysin_2 superimposed to PlyCA, a cysteine/histidine-dependent amidohydrolases/peptidase (CHAP) domain (McGowan et al., 2012). A triad in lysin_2 (Cys486, His566, Asn584) occupies the same position as that of PlyCA catalytic triad (Cys333, His420, Asn438) within the catalytic crevice indicated by a green triangle. Lysin_2 ribbon is rainbow colored, that of PlyCA is grey). **(C)** Ribbon representation of TP-778L Tal CBM_1 (pink) superimposed to the stem domain (yellow) of the Protein O-Linked Mannose N-Acetylglucosaminyl-transferase 1 (Kuwabara et al., 2016) (POMGnT1-Stem, PDB ID 5ggf). The two residues of POMGnT1-Stem Arg129 and Asp179, shown as sticks, are involved in host CWPS binding. The position of these two residues are conserved in CBM_1 (Arg1119 and Asp1174) and are located at the extremity opposite to the trimerization axis (red circle). **(D)** Ribbon representation of TP-778L Tal CBM_2 (pink) superimposed to the junction domain and CBM of phage Tuc2009 BppA baseplate protein (yellow, PDB ID 5e7t). The binding site cavity is indicated by a red circle. **(E)** Ribbon representation of TP-778L Tal CBM_3 (pink) superimposed to the CBM of *C.lostridium thermocellum* cellulase K family 4 (yellow, PDB ID 3p6b). The binding site cavity is indicated by a red circle.

In Moineauviruses (DT1, STP1 and 9851), the Tal N-terminal domains resemble that of prophage MuSo2 from *Shewanella oneidensis* (Table 1). Their well-conserved sequences fold into the typical four sub-domains described above (Supplementary Figure S3). However, in Brussowviruses (SW13 and TP-778L), the Tal N-terminal domains contain insertions between the third and fourth sub-domains (Table 1). Sequences of these domains are quasi-identical (Supplementary Figure S3), and their predicted structures returned the same hit, the Tal from *Listeria monocytogenes* prophage EGD-e (Table 1), using the Dali server. The insertion consists of two modules separated by a short linker (∼10 residues) and connected to sub-domains 3 and 4 *via* long linkers (∼40 residues) (Table 1). These modules from SW13 and TP-778L match closely in sequence and structure (Table 1; Supplementary Figure S3). Therefore, we describe only the N-terminal domain of TP-778L. Module 1 comprises ∼170 residues and is mainly α-helical (Table 1; Figure 3A). A Dali search returned a significant hit with the catalytic domain of the autolysin (Auto) from *L. monocytogenes* (PDB ID 1uto; Lmo1076) (Table 1), an N-acetyl glycosaminidase (Bublitz et al., 2009). Therefore, we named this module “lysin 1.” The catalytic dyad of Auto involves Glu122 and Glu156 on opposite sides of the catalytic crevice (Figure 3A). Of note, the glutamic acid residues Glu 311 and Glu 352 in lysin 1 coincide, at the structural level, with the Auto catalytic residues (Figure 3A). The distances between the two glutamic acid OE1 atoms are 11 Å and 13 Å in Auto and lysin 1, respectively. However, the β−hairpin in Auto, which bears Glu156 of the catalytic dyad and forms a lip of the active site, corresponds to a shorter loop in lysin 1 (Figure 3A). Module 2 comprises ∼150 residues and is formed by a central β-sheet and α-helices on either side (Table 1; Figure 3B). Dali reported a hit with the streptococcal phage lysin PlyCA (Table 1), hence we named this module “lysin 2.” Lysin 2 superimposes well with PlyCA (Figure 3B), a cysteine/histidine-dependent amidohydrolases/peptidase (CHAP) domain, related to papain-like enzymes (McGowan et al., 2012). Notably, a catalytic triad in lysin 2, composed of Cys486, His566, and Asn584, superimposes on the PlyCA catalytic triad, composed of Cys333, His420, Asn438, within the catalytic crevice (Figure 3B).

#### 3.2.2 Tal extensions present different carbohydrate-binding modules combinations

Tal extensions vary from ∼550 residues (DT1) to ∼1,200 residues (TP-778L). In the five phages, a short α-helix immediately follows the N-terminal domain and abuts to a ∼90-residue immunoglobulin (Ig)-like domain (Table 1; Figure 2B). Notably, junctions between N-terminal structural domains and C-terminal extensions were not reliably predicted, therefore we did not join these two domains in our structural models. The Ig-like domain is followed by triple collagen-like linkers of variable lengths, which may be associated with ∼60 amino acid long domains composed of three anti-parallel β-strands, hereafter named “3β domain,” as observed in DT1 and TP-778L (Figures 2A,C). These 3β domains are identified by Dali as being close to half a fibronectin III-like domain, and to part of the junction module of *L. lactis* phage Tuc2009 BppA protein, a CBM-containing component of its adhesion device (Table 1). The next part of the C-terminal extension is formed by different combinations of CBMs, which can be separated by collagen-like linkers and 3β domains as observed in the long Tals of SW13, 9851 and TP-778L (Figure 2A). Our description of these CBMs is based on phage TP-778L since it possesses all the CBMs that are found separately in other phages under study.

The first CBM found along the TP-778L Tal extension, hereafter named CBM_1, was identified by Dali as a stem domain of the Protein O-Linked Mannose N-Acetylglucosaminyltransferase 1 (POMGnT1-Stem) (Kuwabara et al., 2016) (Table 1; Figure 3C). POMGnT1-Stem binds to several monosaccharides such as Glc-β, Man-β, and GlcNAc-β. It has previously been shown that two residues of POMGnT1-Stem, Arg129 and Asp179, play an essential role in saccharide binding (Kuwabara et al., 2016). These two residues are conserved in CBM_1 (Arg1119 and Asp1174) and are located at the opposing end of the trimerization axis (Figure 3C). This CBM is also the first to appear after the N-terminal domain in phage 9851, with quasi-identical sequence and structure to those of TP-778L CBM_1 (Supplementary Figure S3; Table 1). In both phages, CBM_1 is followed by a 3β domain, similar to the Tuc2009 BppA junction module, a collagen-like linker, and another CBM (CBM_2) (Table 1; Figure 2A). This ∼200 amino acid long CBM_2 covers a large part of Tuc2009 BppA adhesion device protein (Legrand et al., 2016), including its CBM domain (Table 1; Figure 3D). Noteworthy, the first CBM found in the Tal of phages STP1 and SW13, just after the N-terminal domain, shares the same fold as that of TP-778L CBM_2, and is therefore also named CBM_2. Interestingly, this CBM_2 and the first CBMs found in the Tal of phages STP1 and SW13, just after the N-terminal domain, share the same fold. However, the CBM_2 in these four phages differ in their sequences. The CBM_2 of phages 9851 and TP-778L possess quasi-identical sequences, and the linker sequence at their N-terminal end is also well conserved. In contrast, the CBM_2 sequences in phages STP1 and SW13 differ from each other, and also from those of 9851 and TP-778L (Supplementary Figure S3). The last ∼160 amino acid long CBM, CBM_3, is connected to CBM_2 *via* a tandem of the collagen-like linker and 3β domain (Table 1; Figure 2A). Noteworthy, the three BppA-like 3β domains of phage TP-778L (3β_3, 3β_4, and 3β_5) and those of DT1, SW13 and 9851 are structurally similar (Table 1). CBM_3 returned a hit with a CBM from *Clostridium thermocellum* cellulase K family 4 using Dali (Table 1; Figure 3E), a domain also found in phages 9851 and SW13. Interestingly, the unique CBM of phage DT1 and the second CBM of phage STP1, named CBM_4, which are in positions equivalent to those of CBM_3 in phages SW13, 9851 and TP-778L along the Tal C-terminal extension and share 76% sequence identity, returned the same hits with the bacteriophage CBA120 tail spike protein and the CBM16 from *Thermoanaerobacterium polysaccharolyticum* ManA (Table 1; Figure 3E). Overall, based on sequence and structural similarities, the remaining CBMs of the Tal extension form two groups. (CBM_3 and CBM_4) The first group comprises CBM_3 of the Moineauviruses DT1 and STP1, while the second group comprises CBM_4 of the Moineauvirus 9851 and the Brussowviruses SW13 and TP-778L (Supplementary Figure S3).

These CBM_3 and CBM_4 located at the Tal distal end are followed in each phage by a long helix (∼90 amino acids) abutting to a β-prism domain formed by 3 × 10 β-strands, except for phage TP-778L in which this domain contains only 3 × 5 β-strands. These domains returned low-confidence and non-functionally relevant hits, using Dali, with various proteins containing anti-parallel β-sheets (Table 1). However, C-terminal β-helices and β-prisms in some phage adhesion devices, such as the *E. coli* phage K1F endo-sialidase CIMCD, the *B. subtilis* phage GA-1 neck appendage protein CIMCD, or the receptor-binding C-terminal domain of phage T5 L-Shaped Tail Fibre, are known to interact with host cell wall receptors, like lipopolysaccharides (Schulz et al., 2010; Garcia-Doval et al., 2015). It is noteworthy that these domains are followed by a chaperone domain, which allows proper folding of the trimeric β-stranded domain and which undergoes autoproteolysis (and therefore absent) in the mature *S. thermophilus* phages (Garcia-Doval et al., 2015).

### 3.3 Predicted structures of receptor-binding proteins

**FIGURE 4:**
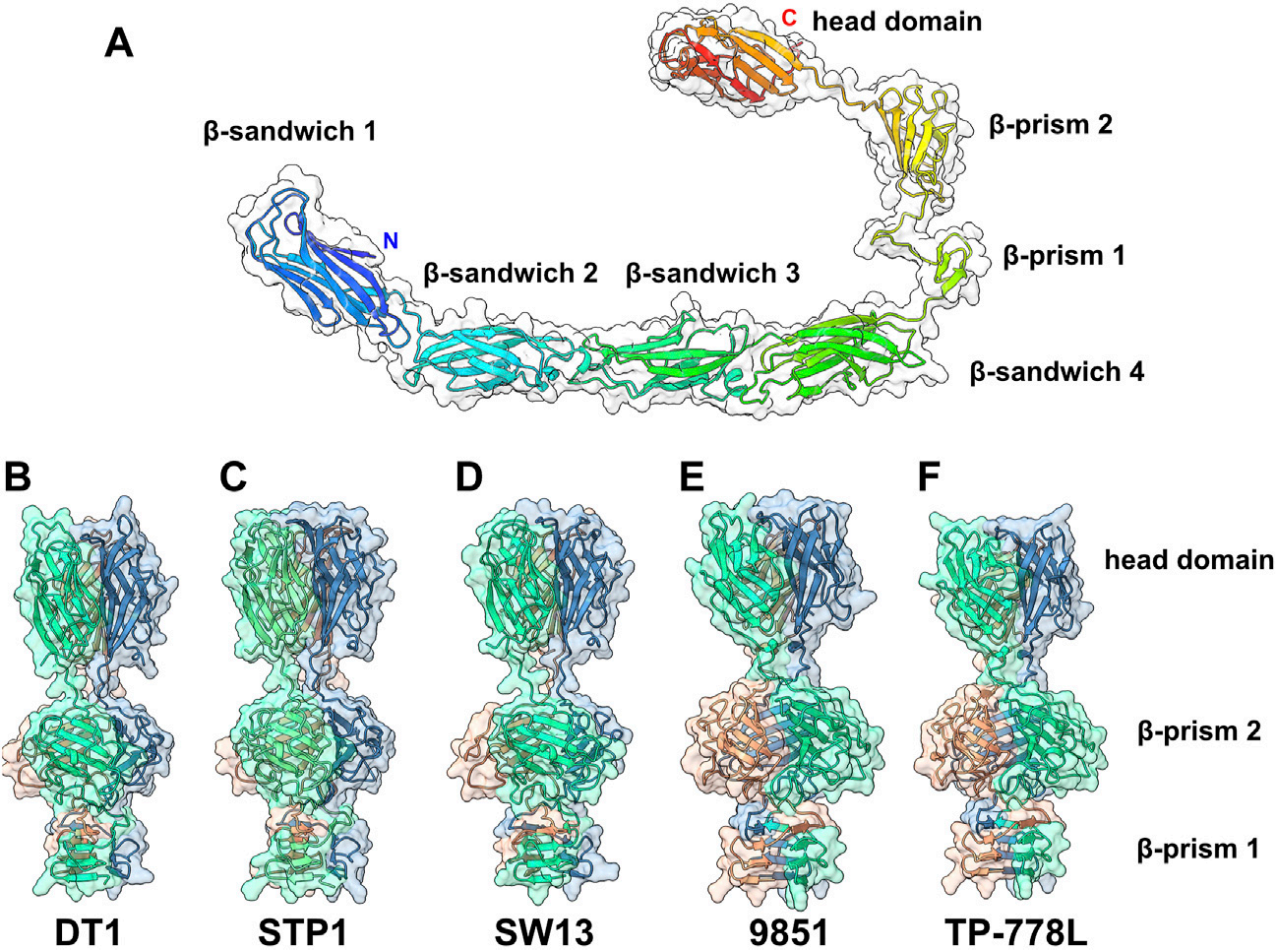
Predicted structure of the RBPs. **(A)** Ribbon and transparent surface representation of TP-778L RBP predicted monomer structure comprising, from the N- to the C-terminal parts, four structural β-sandwiches, two β-prism domains, and the C-terminal head domain. **(B–F)** Ribbon and transparent surface representation of the trimeric β-prisms and C-terminal head domains from the five phages under study (colored by chains).

In the adhesion device-encoding genomic regions of *S. thermophilus* Moineauviruses and Brussowviruses, besides the Dit and Tal pair, we previously identified a third ORF that exhibits the characteristics of a *bona fide* CWPS-specific receptor binding protein (RBP). Structural predictions of the five phage RBPs as monomers identified a linear assembly of seven β-stranded domains (Figure 4A; Supplementary Table S2).

Structure predictions of RBP trimers returned compact assemblies of the last three domains formed of two successive β-prisms of 3 × 3 β-strands and 3 × 4 β-strands, and a β-stranded ternary module resembling the RBP head domain of other Gram positive infecting siphophages (Sciara et al., 2010; Veesler et al., 2012; Dunne et al., 2019) (Figures 4B–F; Supplementary Table S2). However, the first four β-sandwich domains do not assemble together in trimer predictions. Sequence alignment of the five RBPs shows that the β-sandwich and β-prism domains are rather well conserved in *S. thermophilus* phages (Supplementary Figure S4).

Interestingly, Dali analyses of the four β-sandwiches for each phage indicate that their folds differ slightly within the same phage RBP, while β-sandwiches with the same position along the polypeptide chain share similar folds between the different phages (Supplementary Table S2). Lastly, the RBP head sequences are highly divergent and can be grouped in two classes: one includes those of phages DT1, STP1, and SW13, and the other includes those of phages 9851 and TP-778L (Supplementary Figure S4). Consistent with this sequence-based classification, Dali returned hits with the *L. lactis* phage p2 RBP head domain (Sciara et al., 2010) for phages DT1, STP1 and SW13, and with the listerial phage PSA RBP head domain for phages 9851 and TP-778L (Dunne et al., 2019) (Supplementary Table S2).

### 3.4 Comparison of AlphaFold2 predictions with phage nsEM images

**FIGURE 5:**
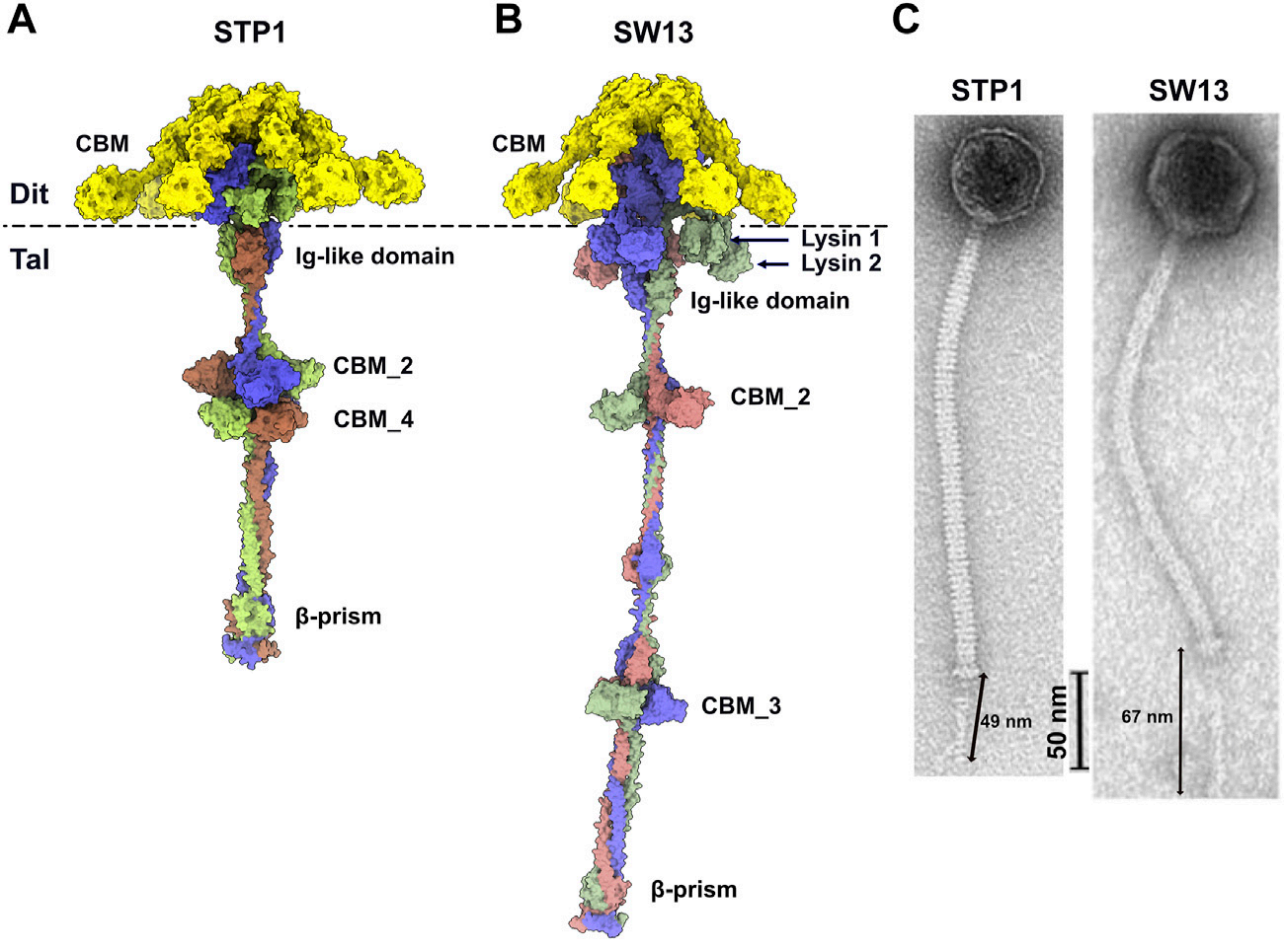
Predicted structure of Dit-Tal assemblies and comparison with nsEM images. **(A,B)** Surface representation of phage STP1 **(A)** and phage SW13 **(B)** Dit-Tal assembly. The Dit hexamer is colored yellow, and the Tal trimer is colored, by chain, green, salmon, and violet. **(C)** nsEM images of phages STP1 and SW13 (Hanemaaijer et al., 2021) with the Dit-Tal assembly length estimated to 49 and 67 nm, respectively.

In order to compare the predicted structures to experimental data, we selected good quality nsEM images from publications of phages STP1 and SW13 and calculated the length of their adhesion devices. The length of the predicted Dit-Tal assembly for STP1 and SW13 was measured, with respective dimensions of 44 and 69 nm (Figures 5A,B). The approximate dimension of the Dit-Tal assembly on phage STP1 nsEM image (Hanemaaijer et al., 2021) (Figure 5C) was estimated to be 49 nm, while that of the same assembly in phage SW13 (Hanemaaijer et al., 2021) was measured at 67 nm (Figure 5C). Since size estimation based on nsEM images is considered difficult, there a in good agreement between our measurements and predictions.

## 4 Discussion

To provide an overview of the structural diversity of dairy streptococcal phage adhesion devices, we have analyzed previously described representative phages with a broad spatio-temporal spread. Phages that infect lactic acid bacteria including *S. thermophilus*, typically exhibit a very narrow host range, often limited to a single or small number of strains. Therefore, the phages analyzed in this study were also selected on the basis of having distinct host strains. Furthermore, these phages belong to one of the two most frequently encountered genera of dairy streptococcal phages, i.e., *Moineauvirus* and *Brussowvirus*.

Our complete AF2-based structural prediction of the three components forming the adhesion device of representative *Moineauvirus* and *Brussowvirus* reveals LEGO-like molecular assemblies of domains with common folds, interspaced by linkers of different lengths. The Dit proteins analyzed in this study all form a core assembling the belt and galectin domains, similar to that observed for other phage Dits (Sciara et al., 2010; Veesler et al., 2010; Veesler et al., 2012) [or an OB-fold in phage T5 (Flayhan et al., 2014)]. Moreover, the Dits’ CBMs exhibit similar folds for the five phages, whose the closest structure in the PDB is that of one of the two CBMs belonging to the Dit of *L. casei* phage J-1 (Dieterle et al., 2017; Goulet, 2022).

The Tals vary very considerably in their length and in the number of modules they possess. Phages DT1, STP1 and 9851 share a common gp27-like N-terminal domain, present in most *Siphoviridae*. In contrast, an insertion is observed in the gp27-like N-terminal domain of phages SW13 and TP-778L. This insertion is projected far away from the trimerization axis and is formed of long linkers bearing two lysin domains, a glycosyl hydrolase and an amino-peptidase, both of which likely target the cell wall peptidoglycan. In the five phages, the N-terminal domain is followed by a short helix connecting it to a conserved Ig-like domain. The five Tal extensions incorporate up to five structural domains formed by a β-sheet of three strands (that we name 3β domain), and between one and three CBMs. It is noteworthy that the last three 3β domains of TP-778L (3β_3−5) resemble a linker domain of phage Tuc2009 adhesion device BppA, and that the CBMs belonging to STP1, SW13, 9851 and TP-778L share the same fold as BppA’s linker and CBM. In contrast with the significant sequence variability associated with Dit CBMs, Tal CBMs are often more conserved at sequence level. While the three Tal CBMs of phages 9851 and TP-778L possess quasi identical sequences, the CBMs of the three other phages differ significantly. This variability is not surprising since these phages bind to different hosts with different saccharide motifs. At the C-terminal end, the five phages share a common structural motif involving a long α-helix and a β-prism. These β-prisms exhibit conserved sequences, with the exception of TP-778L β-prism that is shorter than those of the other four. In contrast with all the other domains of these adhesion devices to which we could assign a function, the functional purpose of these β-prism domains remains mysterious. They likely play a structural role, for example keeping together the Tal trimeric assembly, and/or a host binding function that is observed in some other β-prism domains from phages infecting *E. coli* (Schulz et al., 2010; Garcia-Doval et al., 2015).

The third ORF, which we named RBP due to the structural similarity with lactococcal and listerial RBPs, are formed by a chain of four structural Ig-like domains, followed by three domains found in canonical siphophage RBP. These domains include a tandem of β-prisms followed by a β-sandwich domain resembling closely that of the RBP head domain of lactococcal phage p2 (Spinelli et al., 2006) or listerial phage PSA (Dunne et al., 2019). The RBP C-terminal head domains differ significantly in sequence, which is in agreement with their role in binding host specific CWPS as observed by fluorescence host-binding studies of STP1 and SW13 RBP head domains (Lavelle et al., 2020; Lavelle et al., 2022). We previously identified the position of the six trimeric RBPs of STP1 at the periphery of the Dit-Tal assembly (Lavelle et al., 2020). Although we could not establish connections between RBPs and the adhesion device central core, the pretty well conserved N-terminal β-sandwich domain(s) may be involved in tethering RBPs to the Dit-Tal assembly.

Phages that prevail in dairy fermentation facilities and products are presented with an abundance of potential host cells that facilitate their proliferation. However, the abundance of phages in these fermentations also creates a highly competitive environment and dairy phages have evolved to incorporate multiple CBMs to enhance their ability to initiate contact with potential host strains. The identification of several CBMs within various adhesion device proteins of dairy lactococcal, lactobacilli and streptococcal phages, as well as of phages infecting the wine-making lactic acid bacteria *O. oeni*, highlights the likely advantages of these CBMs for phage infection (Dieterle et al., 2017; Hayes et al., 2018; Hayes et al., 2019; Lavelle et al., 2020; Goulet and Cambillau, 2021; Lavelle et al., 2022). Therefore, it is essential to decipher the presence, diversity, structure and extent of these CBMs and other unique structural features on phage virions.

These AF2 predictions made it possible to perform a precise assignment and analysis of the adhesion device of representative streptococcal phages of the *Moineauvirus* and *Brussowvirus* genera. Thanks to the easy access and user friendliness of AF2 Colab’s NoteBooks, such structural predictions and analyses of phage ORFs constitute a potent and reliable method of phage genomic and functional annotation, and particularly of their often under-annotated adhesion devices.

## Data Availability

The original contributions presented in the study are included in the article/Supplementary Material, further inquiries can be directed to the corresponding authors.
