# Supplementary material for: A structural discovery journey of streptococcal phages adhesion devices by AlphaFold2
Source: Front Mol Biosci. 2022 Aug 19;9:960325. doi: 10.3389/fmolb.2022.960325 (PMC9437275; doi:10.3389/fmolb.2022.960325)
Supplement: Supplementary file 1 [file DataSheet2.PDF]

Supplementary Figure 1. Dit sequence alignments. (a) Alignment of all Dit sequences shown with secondary structures from phage 9851 Dit predicted structure (image produced with Multalin (Corpet, 1988) and ESPript (Gouet et al., 2003). The blue line indicates the CBM insertion. **(b)** Dit sequences can be classified in two groups corresponding to the *Moineauvirus* and *Brussowvirus* genera (alignements produced with Multalin[43]).

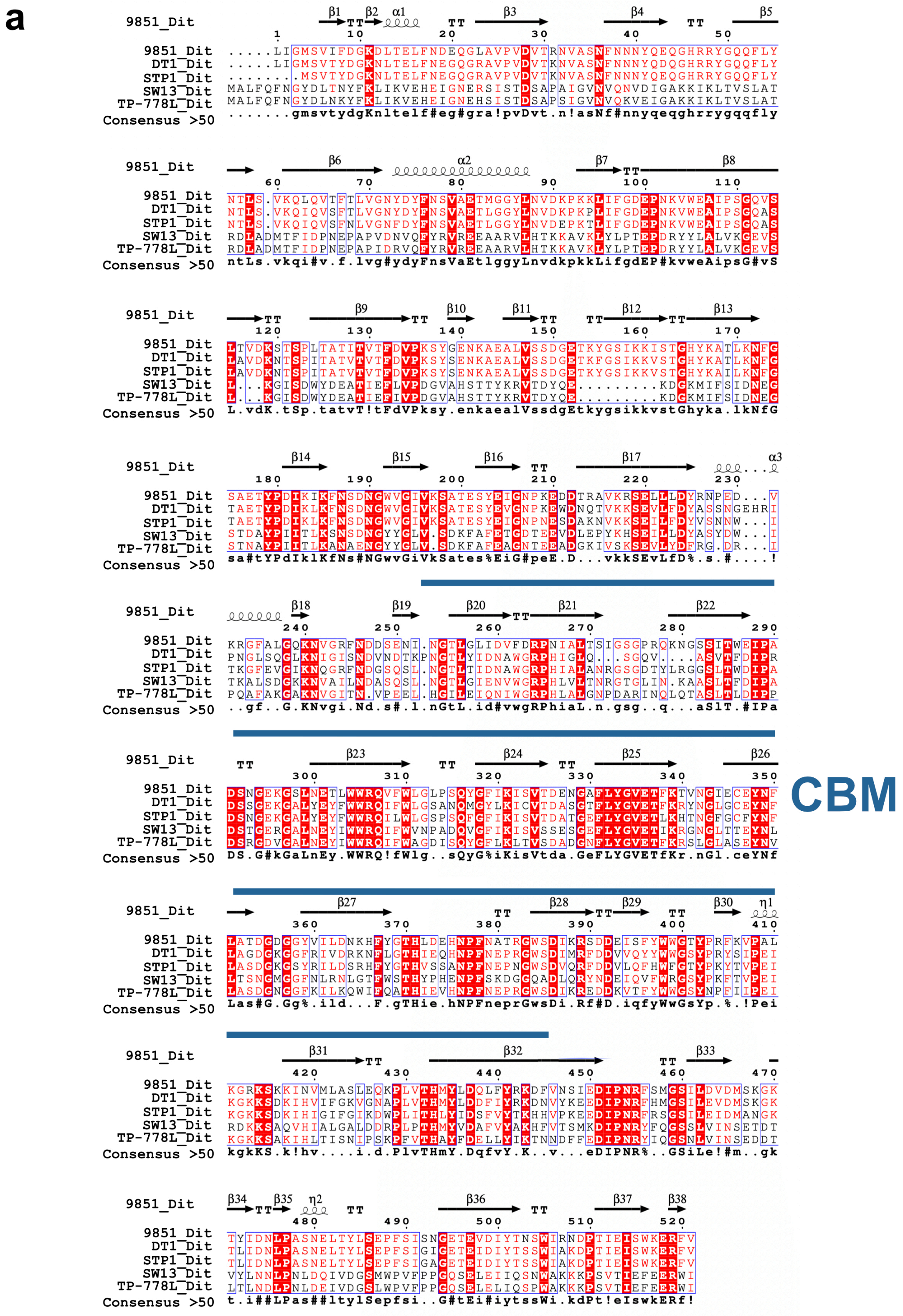

b

- **Dits of phages 9851, DT1, and STP1 (*Moineauvirus* genus)**

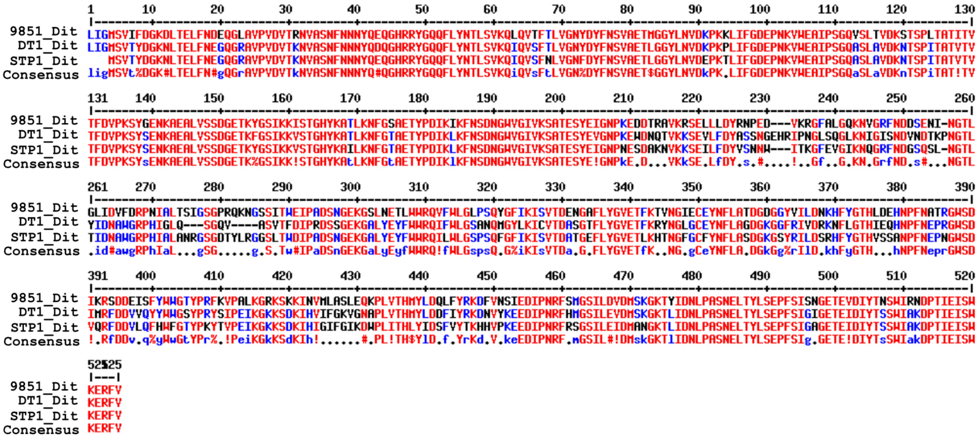

- **Dits of phages SW13 and TP-778L (*Brussowvirus* genus)**

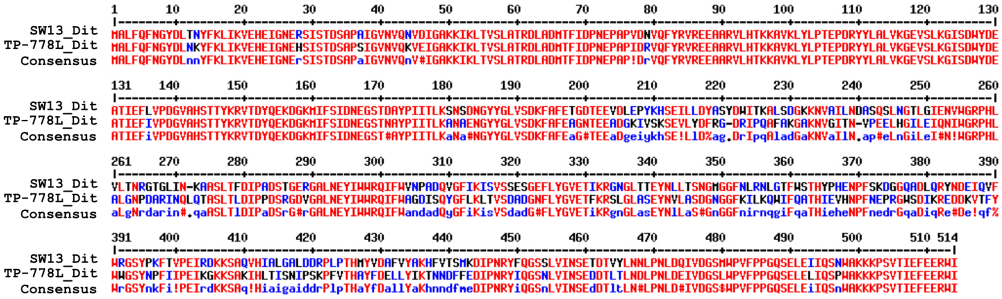

Supplementary Figure 2. Tal sequence alignments. (a) Alignments (Corpet, 1988) of Tal N-terminal sequences (residues 1-520 for *Moineauviruses*, and residues 1-910 *Brussowviruses*). The blue and green lines indicate the four-subdomains assembling the whole gp-27-like structural domain, the orange line indicate lysin domains, and the purple lines indicate Ig-like domains. (**b**) Alignments[43] of CBM sequences identified in Tal extensions.

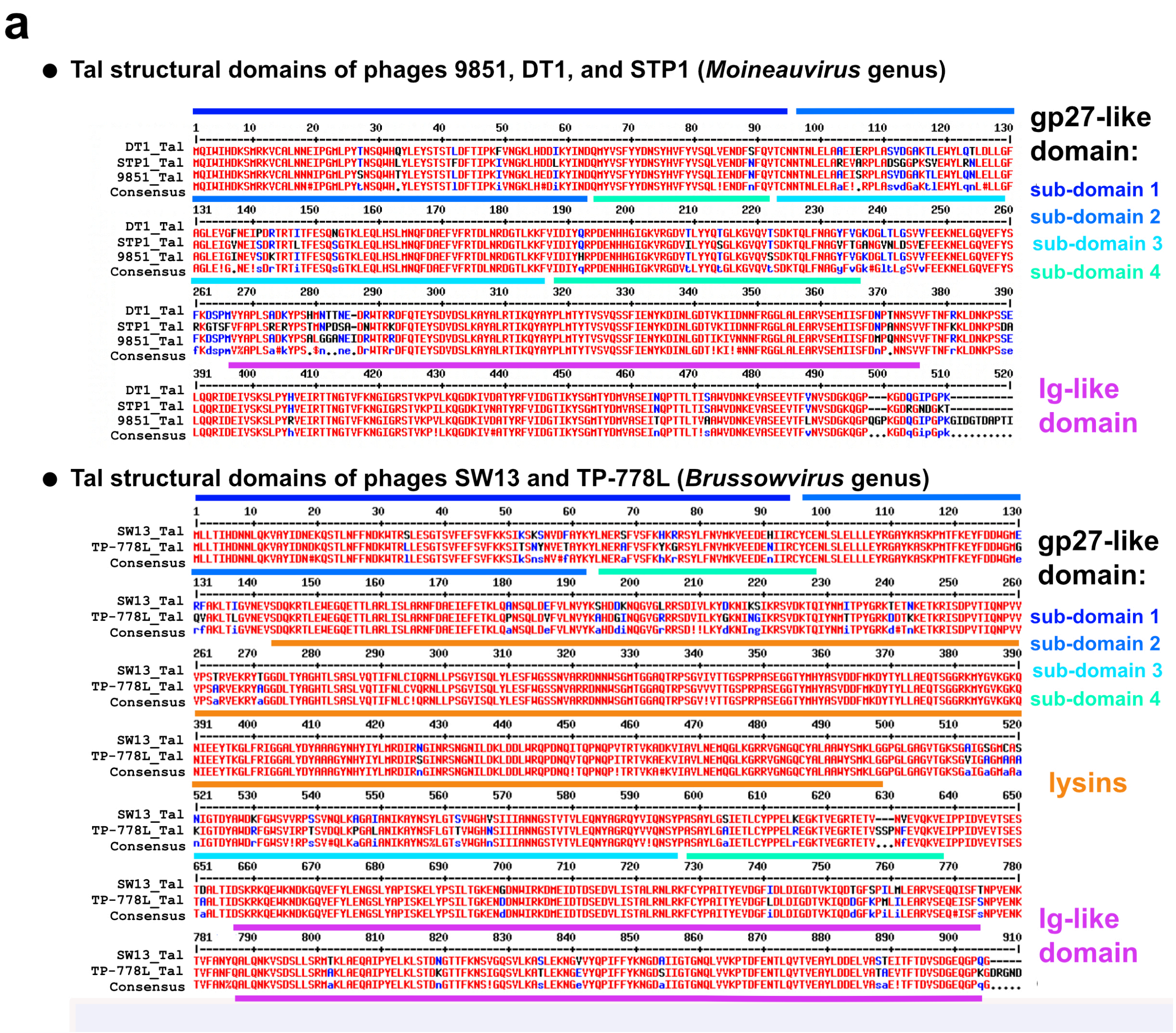

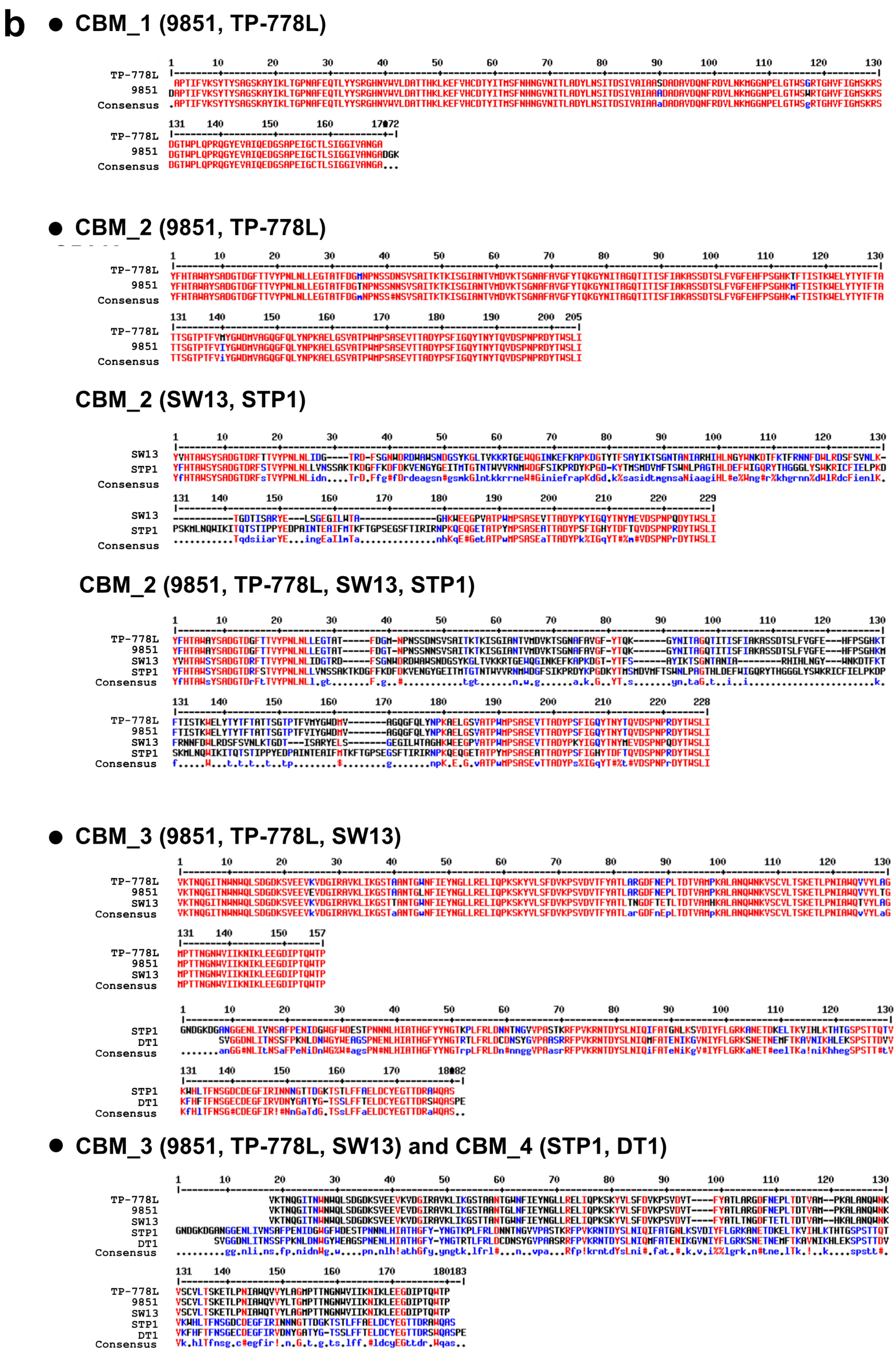
b
CBM_1 (9851, TP-778L)
TP-778L
9851
Consensus
CBM_2 (9851, TP-778L)
TP-778L
9851
Consensus
CBM_2 (SW13, STP1)
SW13
STP1
Consensus
CBM_2 (9851, TP-778L, SW13, STP1)
TP-778L
9851
SW13
STP1
Consensus
CBM_3 (9851, TP-778L, SW13)
TP-778L
9851
SW13
Consensus
STP1
DT1
Consensus
CBM_3 (9851, TP-778L, SW13) and CBM_4 (STP1, DT1)
TP-778L
9851
SW13
STP1
DT1
Consensus

Supplementary Figure 3. RBP sequence alignments. Alignment (Corpet, 1988) of all RBP sequences shown with secondary structures from phage STP1 RBP predicted structure. The different domains are indicated with colored lines.

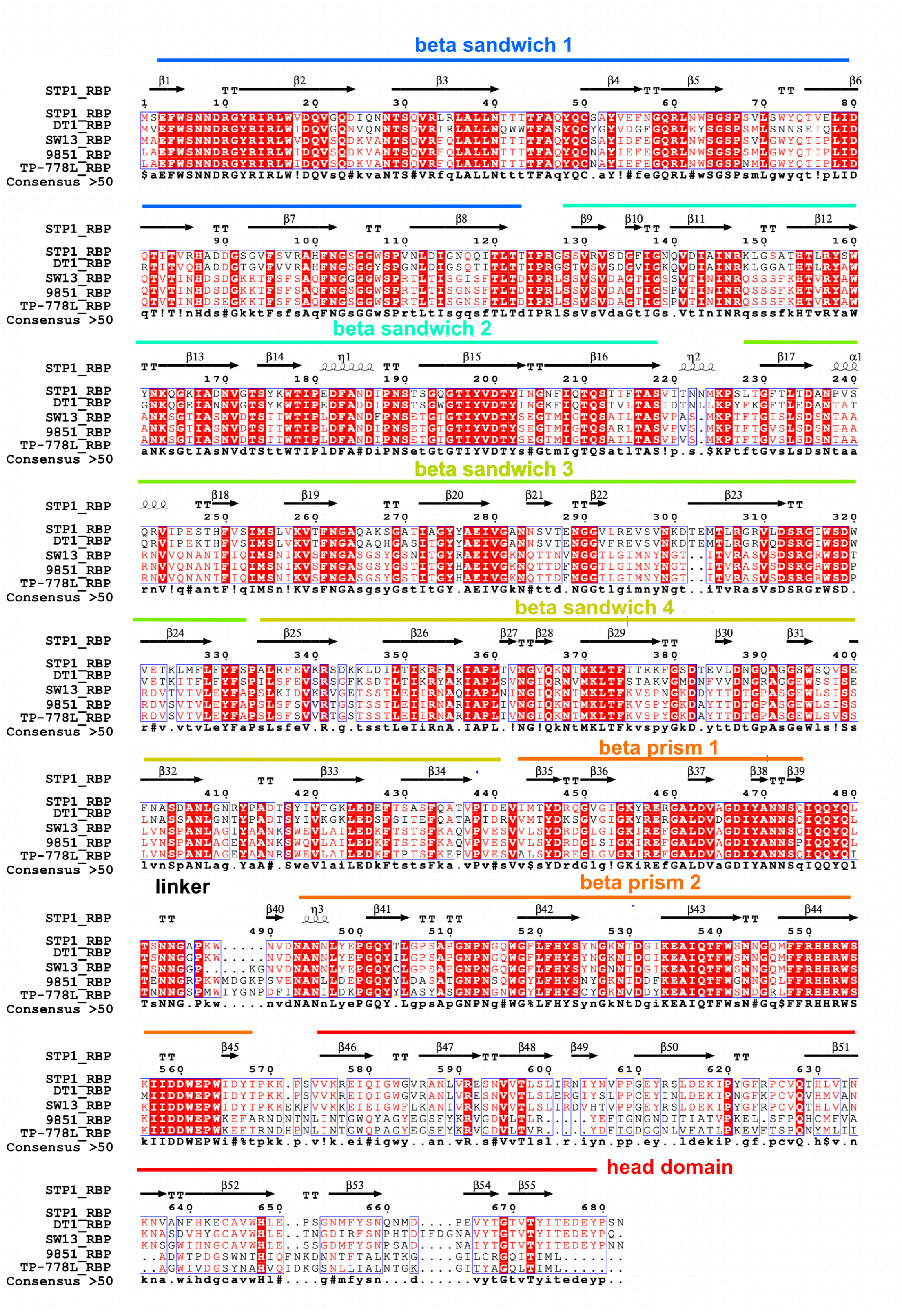

Supplementary Figure 4. pLLDT values of (a) Dit and RBP structure predictions and (b) Tal structure predictions. pLDDT values for each residue along protein sequences are plotted.

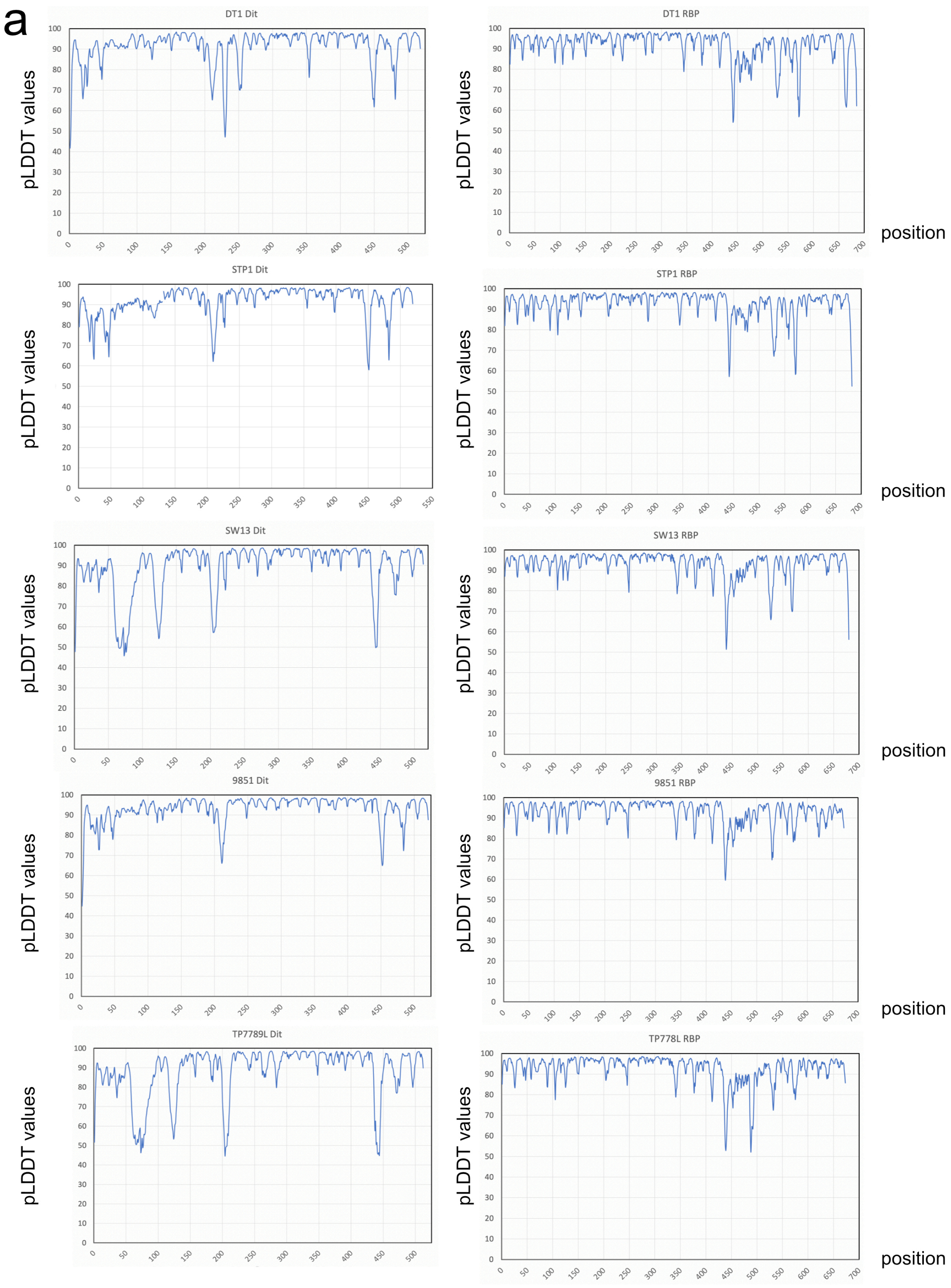

b

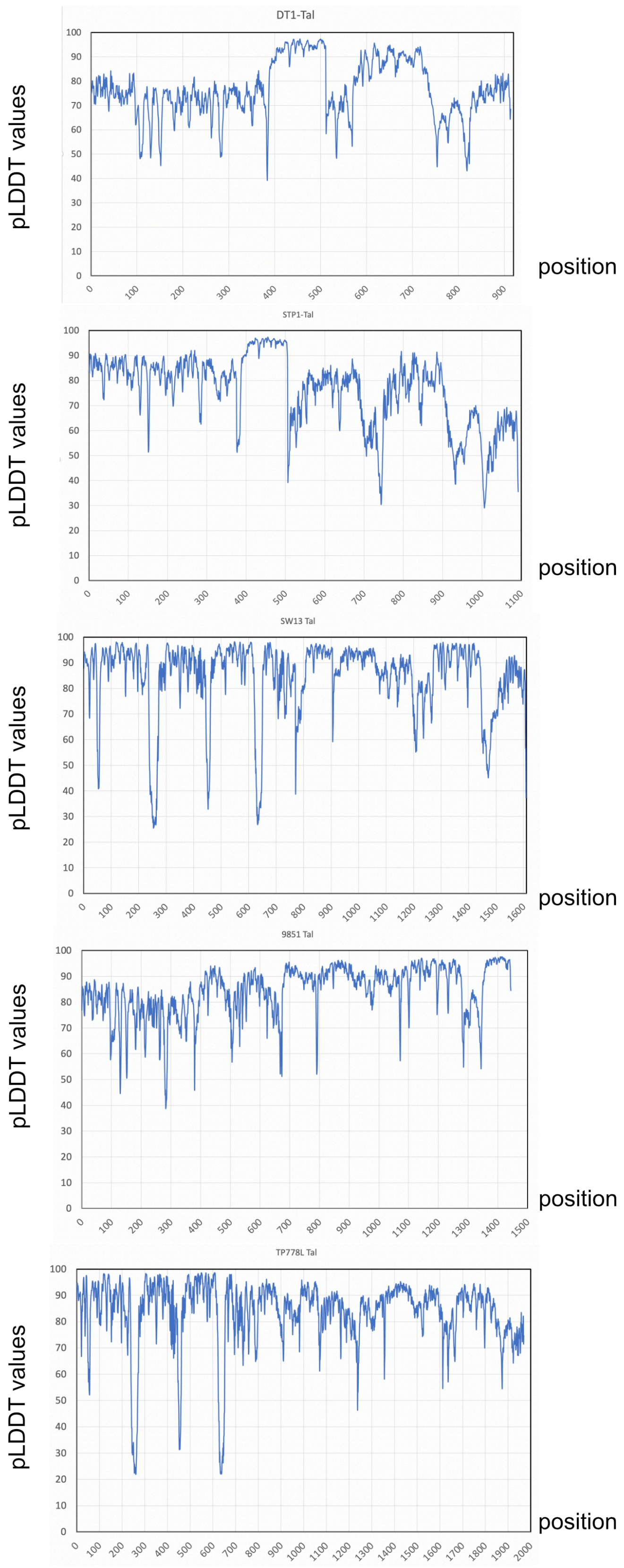
DT1-Tal
pLDDT values
position
STP1-Tal
pLDDT values
position
SW13 Tal
pLDDT values
position
9851 Tal
pLDDT values
position
TP778L Tal
pLDDT values
position
