# Supplementary material for: A structural discovery journey of streptococcal phages adhesion devices by AlphaFold2
Source: Front Mol Biosci. 2022 Aug 19;9:960325. doi: 10.3389/fmolb.2022.960325 (PMC9437275; doi:10.3389/fmolb.2022.960325)
Supplement: Supplementary file 3 [file DataSheet1.PDF]

Supplementary Table 1. Dit domain boundaries in predicted structures (M: *Moineauvirus;* B: *Brussowvirus*; § : PDB ID; Z score).

	**DT1 (M)**	Dali hit§	**STP1 (M)**	Dali hit§	**SW13 (B)**	Dali hit§	**9851 (M)**	Dali hit§	**TP-778L (B)**	Dali hit§
Belt/ galectin part 1	1-206		1-203		1-199		1-206		1-199	
linker	207-258		204-246		200-242		207-259		200-246	
CBM	259-440	5ly8;17.4	247-440	5ly8;19.3	243-435	5ly8;22.9	250-443	5ly8;23.1	247-434	5ly8;22.0
linker	441-457		441-457		436-452		444-460		435-451	
Belt/ galectin part 2	458-518		458-518		453-513		461-521		452-512	
Belt/galectin all	1-206/ 458-518	2x8k;12.6	1-203/ 458-518	2x8k;12.5	1-199/ 453-513	2x8k;20.2	1-206/ 461-521	2x8k;13.2	1-199/ 452-512	2x8k;12.4

Supplementary Table 2. RBP domain boundaries in predicted structures (M: *Moineauvirus;* B: *Brussowvirus*; § : PDB ID; Z score).

	**DT1 (M)**	Dali hit§	**STP1 (M)**	Dali hit§	**SW13 (B)**	Dali hit§	**9851 (M)**	Dali hit§	**TP-778L (B)**	Dali hit§
β-sandwich 1	1-121		1-122	6zlx;9.9	1-120		1-120		1-120	6zlx;9.3
linker	121-128		123-128		121-128		121-128		121-128	
β-sandwich 2	129-218		129-219	6mvh;9.6	129-218		129-218		129-218	6mvh;9.5
linker	219-229		220-229		219-228		219-228		219-228	
β-sandwich 3	230-328		230-328	2y72;10.3	229-325		229-325		229-325	2y72;10.5
linker	329-333		329-234		326-330		326-330		326-330	
β-sandwich 4	334-437		335-437	2hvb;9.0	331-434		331-434		331-434	2hvb;8.8
linker	438-441		438-440		435-440		435-440		435-440	
β-prism 1	442-464		441-471		441-461		441-461		441-461	
linker	465-491		472-500		462-497		462-502		462-502	
β-prism 2	492-552		501-553		498-549		503-569		503-569	
linker	553-573		554-573		550-572		570-575		570-576	
Head domain	574-685	2bse;13.3	574-682	2bse;13.4	573-680	2bse;13.6	576-672	6r5w;11.0	577-673	6r5w;10.3
